# Methicillin-Resistant *Staphylococcus aureus*: The Magnitude and Risk Factors among Patients Admitted to Tikur Anbessa Specialized Hospital, Addis Ababa, Ethiopia

**DOI:** 10.1155/2021/9933926

**Published:** 2021-10-27

**Authors:** Tewodros Tamire, Temesgen Eticha, Temesgen Bati Gelgelu

**Affiliations:** ^1^Tikur Anbessa Specialized Hospital, Addis Ababa, Ethiopia; ^2^Department of Medical Laboratory Sciences, College of Health Sciences and Medicine, Wolaita Sodo University, Sodo, Ethiopia; ^3^School of Public Health, College of Health Sciences and Medicine, Wolaita Sodo University, Sodo, Ethiopia

## Abstract

**Background:**

In healthcare facilities, a gradual increase in methicillin-resistant *Staphylococcus aureus* (MRSA) infections has been seen over the past 2 decades. Similarly, it has been responsible for the most frequent and invasive pathogens associated with admitted patient infection. Currently, it is considered an urgent threat to public health and classified as one of the top-priority antimicrobial-resistant pathogens. This study aimed to determine the magnitude and associated risk factors of MRSA infection among admitted patients.

**Methods:**

A facility-based cross-sectional examination was led on 413 patients admitted to Tikur Anbessa Specialized Hospital from January 2018 to January 2019. A convenient sampling technique was used. Clinical specimens of pus and blood were collected from admitted patients who developed the infection after 48 hours of admission. Gram stain, culture media preparations, and biochemical tests were conducted to identify and isolate the causative agent. *Staphylococcus aureus (S. aureus)* were identified as MRSA strains after having a zone of inhibition less than or equal to 21 mm to the cefoxitin (30 ug) disc. Bivariate and multivariable logistic regression analyses were computed. The odds ratio, along with 95% CI, was estimated to identify associated risk factors for MRSA infection.

**Results:**

Out of 413 collected specimens, 38.7% had coagulase-positive *S. aureus* of which 35.6% (95% CI: 28.2%–43.0%) were MRSA. Being within the age group of 19–29 years and 30–39 years with AOR = 5.02 and 95% CI: 1.24–20.35 and AOR = 6.65 and 95% CI: 1.78–24.78, respectively, admitting in the hematology ward and the pediatric ward with AOR = 7.80 and 95% CI: 1.82–33.49 and AOR = 10.54 and 95% CI: 1.78–62.42, respectively, and experiencing poor prognosis with AOR = 10.97 and 95% CI: 4.57–26.36 were significantly associated with MRSA infection. *Conclusion and Recommendation.* The significant magnitude of MRSA was found among patients admitted to this hospital. Therefore, identified risk factors should be considered when executing hospital-acquired infection prevention programs. We also suggest that healthcare providers should consider the identified risk factors while prescribing the antibiotic.

## 1. Introduction

Methicillin-resistant *Staphylococcus aureus* (MRSA) is a type of staph bacteria that is potentially dangerous and resistant to certain antibiotics. MRSA has high adaptability, colonization capability, and the ability to develop resistance. This makes it a virulent and difficult-to-treat microorganism. It is persistently emerging with new strains often resulting in sustained epidemics. MRSA is now considered an urgent threat to public health and classified as one of the top-priority antimicrobial-resistant pathogens [1, 2].

In healthcare facilities, a gradual increase in MRSA infections has been seen over the past 2 decades. Lately, it has been responsible for the most frequent and invasive pathogens associated with patients' infections. Due to its new virulence factors, this organism can also cause necrotizing and frequently lethal pneumonia [3].

MRSA is usually spread by direct contact with an infected wound or from contaminated hands, usually those of healthcare providers, then causes severe problems, and leads to sepsis or death to patients admitted in healthcare facilities. Evidence has been noted that antibiotic resistance could be an outcome of inappropriate use of antibiotics and/or nonadherence to institutional guidelines. Likewise, the regulatory system of healthcare facilities of the developing countries is weak due to a lack of adequate resources. This gap creates a challenge for hospital managers during the planning of appropriate antibiotics and on the physician in prescribing appropriate antibiotics for their patient, which in turn result in high patient's financial burden of hospitalization. This problem is becoming serious in a setting where second-line antibiotics are expensive and even not accessible, such as in Ethiopia [1, 4, 5].

Different studies assessed the magnitude of MRSA and its risk factors in Ethiopia. The studies reported a distinct magnitude of MRSA which is 49.7%, 2.4%, and 17.5%. Besides, the evidence showed confounded risk factors, used specimens from nasal/throat/wound/ear, and studied some wards [6–9]. However, relative information from the hematology ward, pediatric ward, and blood specimen should not be undervalued. Therefore, we determined the magnitude and associated risk factors for MRSA by studying all wards and using both pus and blood specimens.

## 2. Materials and Methods

### 2.1. Study Design, Setting, and Population

A facility-based cross-sectional study was conducted from January 2018 to January 2019 in Tikur Anbessa Specialized Hospital, Addis Ababa, Ethiopia. The hospital has 800 beds and provides referral services of the OPD, surgery, family planning, ART, laboratory, pharmacy, ANC, oncology, ENT, delivery, and neonatal care for the community in and outside the capital city. All patients admitted to this hospital were the source population. All patients admitted to the ICU, the surgery, the hematology, the pediatric, the orthopedics, and gynecology wards who were suspected by the physician to bacterial nosocomial infection after 48 hours of admission or patients with a sign and symptom of bacterial nosocomial infection in the hospital during the study period were the study population.

### 2.2. Sample Size Determination and Sampling Technique

All patients admitted to the ICU, the surgery, the hematology, the pediatric, the orthopedics, and the gynecology wards from January 2018 to January 2019, 413 patients, were included. Besides, the convenience sampling technique was used because the patients that were admitted at the time of data collection were selected.

### 2.3. Specimen Collection and Examination

After providing informed consent, the clinical specimen of pus and blood was collected from patients admitted in the ICU, the surgery, the hematology, the pediatric, the orthopedics, and the gynecology wards who developed an infection after 48 hours by employing standard microbiological procedures. All collected specimens were transported to the microbiology laboratory of the hospital with minimum delay for culture and susceptibility tests. About 2.5–5 ml of the blood specimen was inoculated into aerobic 30 ml BACT/ALERT PF Plus pediatric bottles of the blood-to-broth ratio of 1 : 10–1 : 30, and two bottles of blood samples were collected per patient and incubated aerobically at 37°C for 5 days. The incubation was followed strictly for bacterial growth alarm. Wound specimens aseptically collected from the appropriate site of infection were cultured on both mannitol salt agar and blood agar. Gram stain and then the biochemical test were conducted consecutively to isolate the causative agents.

### 2.4. Culture and Gram Staining

In accordance with Clinical Laboratory Standards Institute guidelines (CLSI) (10), clinical specimens were inoculated onto the blood agar base (Oxoid, Basingstoke, Hampshire, England) to which 5% sheep blood was added and mannitol salt agar (Oxoid, Basingstoke, Hampshire, England) by using the streaking method. Inoculated plates were incubated at 35–37°C for 18 to 24 hrs aerobically. Bacterial colonies showing typical characteristics of *S. aureus* (i.e., beta-hemolytic on blood agar and colonies with golden yellow pigmentation on mannitol salt agar) were subjected to subculture onto basic media, Gram stain, catalase, coagulase, and Pastorex staph extract. These catalase, coagulase, Pastorex, and Gram-positive bacteria appearing in grape-like clusters were considered as *S. aureus* [8, 10].

### 2.5. MRSA Strain Detection

Cefoxitin (30 ug) susceptibility test was carried out by the Kirby–Bauer disc diffusion method as per CLSI guidelines on Mueller–Hinton agar (Oxoid, Basingstoke, England) for cefoxitin. The antibiotic disc of cefoxitin was placed after 15 min of inoculation to Mueller–Hinton agar and incubated for 24 h at 35–37°C. The diameter of the zone of inhibition around the disc was measured using sliding metal calipers. Strains of *S. aureus* were identified as MRSA after having a zone of inhibition less than or equal to 21 mm to the cefoxitin disc [10].

### 2.6. Data Quality Assurance

All demographic and risk factors related to data were collected from the patient itself or from the caretaker in the hospital. A pretest was done in another hospital ward that provides similar services, and then based on the finding of a pretest, necessary corrections were made. The completeness, readability, and clearness of questionnaires were checked.

To assure the quality of data related to sample collection and examination, data collectors and supervisors with a health background were trained and assigned. Besides, the performance of the equipment was checked during the pretest, using standards and daily quality control. Furthermore, visual examinations of breaks in media or plastic Petri dishes, inconsistent fill, hemolysis, freezing, air pockets, and contamination were made. Furthermore, the standard operating procedures (SOP) were strictly followed verifying that the media meet expiration date and quality control parameters per CLSI standards. Furthermore, the standard strains (ATCC25923) were used to control the quality of the new lot before testing *S. aureus*. Moreover, collected samples were sent immediately to the laboratory with a minimum delay for analysis. Finally, culture results were double-checked to ensure the accuracy of the reading.

### 2.7. Data Management and Analysis Procedure

Data were entered into Excel and then imported to SPSS V25 for processing and analysis. Cleaned data were diagnosed for collinearity using the variance inflation factor before further statistical analysis. Descriptive statistics such as frequencies and proportions were performed to show the distribution of outcomes among the study participants. The crude association between independent and dependent variables was assessed using a bivariate analysis at a *P* value less than 0.25. Finally, associated risk factors for outcome variables were identified using multivariate analysis at a *P* value less than 0.05. The Hosmer and Lemeshow goodness-of-fit test result indicates that the final model was fit with *P* value = 0.623. Besides, the omnibus tests of model coefficients indicate that the model with predictors is better off (*P* value = 0.0001).

## 3. Results

### 3.1. Magnitude of MRSA among Admitted Patients

Out of 413 collected specimens, 38.7% had coagulase-positive *S. aureus* of which 35.6% (95% CI: 28.2%–43.0%) were MRSA. The higher burden of MRSA was found among males and between 30 and 39 age group patients, which was 39.3% (35/89) and 50.0% (14/28), respectively. From the total isolates identified, MRSA was highest in the hematology wards (50.0% (16/32)), and the least was observed in the orthopedics (11.8% (4/34)). Of the total *S. aureus* bloodstream infection, more than one-third (38.2% (26/68)) were identified as MRSA. In this study, MRSA was responsible for more than half (57.6% (34/59)) of the recoveries ended with complications, while it was responsible for eight out of nine deaths; the difference was statistically significant (Table 1).

### 3.2. Factors Associated with Methicillin-Resistant *Staphylococcus aureus*

As presented in Table 2, a crude association between admitted patients' characteristics and MRSA was assessed first by using bivariate logistic regression analysis at a cut point of *P* value less than 0.25. In the meantime, admitted patients' characteristics such as the age of the admitted patients, the type of wards, and prognosis of the nosocomial infection had a *P* value less than 0.25, which were selected as candidate variables for multivariable logistic regression analysis. Similarly, in multivariable logistic regression analysis, an independent association between the candidate variables and MRSA was further assessed at a cut point of *P* value less than 0.05. Accordingly, the result of multivariable logistic regression analysis revealed the following factors statistically associated with acquiring MRSA infection: being in the age group of 19–29 and 30–39 years, being admitting in the hematology and pediatric ward, and experiencing poor prognosis (Table 2).

Table 2 also presents multivariable logistic regression analysis results; admitted patients who were found within the age group of 19–29 years and 30–39 years had 5 times (AOR = 5.02; 95% CI: 1.24–20.35) and more than 6 times (AOR = 6.65; 95% CI: 1.78–24.78) a higher risk of acquiring MRSA than those who aged below or equal to 18 years, respectively. Patients who were admitted to the hematology ward and the pediatric ward had 8 times (AOR = 7.80; 95% CI: 1.82–33.49) and more than 10 times (AOR = 10.54; 95% CI: 1.78–62.42) a higher risk of acquiring MRSA than those admitted to the orthopedics ward, respectively. The relative probability of experiencing a poor prognosis was 11 times (AOR = 10.97; 95% CI: 4.57–26.36) higher among patients who acquired MRSA than those who acquired MSSA.

## 4. Discussion

In the current study findings, the overall magnitude of coagulase-positive *S. aureus* isolation rate is in line with the findings from Debre Markos Hospital (39.7%), higher than from Mekele (32.5%), Madagascar (11%), and Yekatit 12 hospitals (14.3%) and less than reports from Pakistan (55%) [6, 8, 11, 12]. The higher findings in this study might be related to the type of specimen which was used by this study, while nasal swab was used by other studies that may be attributed to the observed difference. Besides, this variation in magnitude may be as a result of several factors such as strength of hospital-acquired infection prevention and control committee, healthcare supplies and facilities available in the particular hospital, and rationale antibiotic usage which varies from hospital to hospital [13].

Out of the 160 *S. aureus* isolates, more than one-third ((35.6%), 95% CI: 28.2%, 43.0%) were MRSA. The magnitude of MRSA in this study is consistent with the study findings reported from Moi Teaching and Referral Hospital of Kenya (37%) [14] and from five tertiary hospitals of South Africa (30.9%) [15], but by far less than the study reported from Debre Markos Hospital (49.7%) [6] and Bangladesh (72%) [16] and incomparably higher than findings of 2.4% in Mekele [7] and Yekatit 12 Hospitals (17.5%) [8]. This finding was also higher than other studies conducted in Madagascar (1.3%) and Italy (1.15%) [12, 17]. This variation may due to differences in the age groups, study subjects, and type of used specimen which could be attributed to the observed difference between the studies. In addition, strong hospital-acquired infection prevention and drug monitoring policy differences might also be contributed to the difference.

This significant magnitude of MRSA in the study setting might be related to the inappropriate use of antibiotics and/or nonadherence to institutional guidelines [4, 5]. Therefore, we suggest that every effort should be made to prevent the spread of MRSA; most importantly, early diagnosis and appropriate management of the infected patients are required. Besides, it would be better if the hospital managers look over the existing infection control practices and adherence to the institutional guideline for the use of antibiotics and patient care.

After controlling for confounding factors, the risk of acquiring MRSA infection was higher among the patients who were found within the age group of 19–29 years and 30–39 years, admitted to the hematology ward and pediatric ward, and those who experienced the poor prognosis compared to their counterparts.

In disagreement with a study that reported antibiotics targeting cell wall synthesis do not display an age-dependent pattern and have a similar degree of resistance across all age classes [18], this study found that admitted patients who were found within the age group of 19–29 years and 30–39 years had five times and more than six times higher risk of acquiring MRSA than those who were found below or equal to 18 years' age group, respectively. This difference might be related to the important patient factors that could influence infection acquisition probability such as the status of the immunity, underlying disease condition, and diagnostic and therapeutic intervention differences. This finding suggests that healthcare providers should consider patient age as a risk factor while prescribing this type of antibiotic which might also help in preventing the possibility of future resistance.

According to the finding of this study, patients who were admitted to the hematology ward had nearly eight times higher risk of acquiring MRSA than those who were admitted to the orthopedics ward. This might be related to the fact that needle insertion to draw blood samples and transfusion of unscreened blood and blood products could increase the opportunity of inoculating the organisms, possibly antibiotic-resistant strains. This result indicates the need of establishing strong infection control strategies that help to prevent the spread of MRSA among patients admitted to this ward.

Besides, patients who were admitted to the pediatric ward had more than ten times higher risk of acquiring MRSA than those who were admitted to the orthopedics ward. This finding was supported by the study finding from South Africa that reported neonates were at the highest risk of developing hospital-related MRSA infection [15]. Thus, the need to control MRSA infection is required in neonates that have breaches in their bodies as they contribute the most to the burden of the disease and are likely to have unfavorable outcomes.

Furthermore, this study revealed that the relative probability of experiencing a poor prognosis was eleven times higher among patients who acquired MRSA than those who acquired MSSA. This might be due to the nature of the MRSA strain that has the capability of resisting the effects of commonly prescribed antibiotics. This could create favourable conditions for the infection to disseminate and become severe. Therefore, this finding suggests that attention should be paid to prevent the poor prognosis of the infection by early diagnosis of MRSA, and most importantly, testing the efficacy of the antibiotic is required before use. In this study, as limitations, molecular-level MRSA strain identification was not done due to the lack of access to facilities such as PCR, extraction kit, and detection kit. Besides, the nature of the study design used in this study is incapable of establishing a temporal relationship. Therefore, we strongly recommend other researchers to do molecular-level strain identification and use stronger study designs.

## 5. Conclusion and Recommendation

The significant magnitude of MRSA was found among patients admitted to this hospital. Being within the age group of 19–29 years and 30–39 years, admitting to the hematology ward and the pediatric ward, and experiencing poor prognosis were significantly associated with MRSA infection. Therefore, identified risk factors should be considered when executing hospital-acquired infection prevention programs. We also recommend establishing strong infection control strategies to prevent the spread of MRSA among patients admitted to this hospital, particularly to the hematology and the pediatric ward. Furthermore, this finding suggests that healthcare providers should consider the identified risk factors while prescribing the antibiotic.

## Figures and Tables

**Table 1:** Magnitude of MRSA among patients admitted to Tikur Anbessa Specialized Hospital, Addis Ababa, Ethiopia, from January 2018 to January 2019 (*n* = 160).

Variables		MRSA			
		Yes		No	
		*N* (57)	% (35.6)	*N* (103)	% (64.4)
Age	≤18	17	27.0	46	73.0
	19–29	9	42.9	12	57.1
	30–39	14	50.0	14	50.0
	40–49	7	31.8	15	68.2
	≥50	10	38.5	16	61.5

Sex	Male	35	39.3	54	60.7
	Female	22	31.0	49	69.0

Ward	Orthopedics	4	11.8	30	88.2
	Gynecology	4	33.3	8	66.7
	Hematology	16	50.0	16	50.0
	Surgery	15	40.5	22	59.5
	Pediatrics	8	40.0	12	60.0
	Intensive care unit	10	40.0	15	60.0

Specimen	Blood	26	38.2	42	61.8
	Pus	31	33.7	61	66.3

Current or past history of IV lines' usage	Yes	55	36.2	97	63.8
	No	2	25.0	6	75.0

Presence of chronic wound infections	Yes	20	35.7	36	64.3
	No	37	35.6	67	64.4

Contact with pets	Yes	51	36.7	88	63.3
	No	6	28.6	15	71.4

History of blood transfusion	Yes	38	35.5	69	64.5
	No	19	35.8	34	64.2

Last six-month admission history	Yes	40	34.8	75	65.2
	No	17	37.8	28	62.2

Hospital admission length in weeks	≤ a week	52	37.1	88	62.9
	> a week	5	25.0	15	75.0

Types of nosocomial infections	Bloodstream infection	26	38.2	42	61.8
	Surgical site infection	31	33.7	61	66.3

Prognosis of the infections	Recovery without complications	15	16.3	77	83.7
	Recovery with complications	34	57.6	25	42.4
	Death	8	88.9	1	11.1

**Table 2:** Factors associated with MRSA among patients admitted to Tikur Anbessa Specialized Hospital, Addis Ababa, Ethiopia, from January 2018 to January 2019 (*n* = 160).

Variables		MRSA (35.6%)		
		Yes, *n* = 57	Cor (95% CI)	AOR (95% CI)
Age	≤18	17 (27.0%)	1.00	1.00
	19–29	9 (42.9%)	2.03 (0.73, 5.67)	5.02 (1.24, 20.35)^*∗*^
	30–39	14 (50.0%)	2.71 (1.07, 6.83)	6.65 (1.78, 24.78)^*∗*^
	40–49	7 (31.8%)	1.26 (0.44, 3.63)	2.07 (0.52, 8.23)
	≥50	10 (38.5%)	1.69 (0.64, 4.45)	3.33 (0.93, 11.91)

Sex	Male	35 (39.3%)	1.00	
	Female	22 (31.0%)	0.69 (0.36, 1.34)	

Ward	Orthopedics	4 (11.8%)	1.00	1.00
	Gynecology	4 (33.3%)	3.75 (0.77, 18.39)	3.26 (0.53, 20.10)
	Hematology	16 (50.0%)	7.50 (2.14, 26.24)	7.80 (1.82, 33.49)^*∗*^
	Surgery	15 (40.5%)	5.11 (1.49, 17.54)	3.60 (0.84, 15.42)
	Pediatrics	8 (40.0%)	5.00 (1.27, 19.76)	10.54 (1.78, 62.42)^*∗*^
	ICU	10 (40.0%)	5.00 (1.34, 18.62)	2.34 (0.51, 10.65)

Specimen	Blood	26 (38.2%)	1.00	
	Pus	31 (33.7%)	0.82 (0.43, 1.58)	

Current or past history of IV lines' usage	Yes	55 (36.2%)	1.70 (0.33, 8.72)	
	No	2 (25.0%)	1.00	

Presence of chronic wound infections	Yes	20 (35.7%)	1.01 (0.51, 1.98)	
	No	37 (35.6%)	1.00	

Contact with pets	Yes	51 (36.7%)	1.45 (0.53, 3.97)	
	No	6 (28.6%)	1.00	

History of blood transfusion	Yes	38 (35.5%)	0.99 (0.49, 1.96)	
	No	19 (35.8%)	1.00	

Last six-month admission history	Yes	40 (34.8%)	0.88 (0.43, 1.79)	
	No	17 (37.8%)	1.00	

Hospital admission length in weeks	≤ a week	52 (37.1%)	1.00	
	> a week	5 (25.0%)	0.56 (0.19, 1.64)	

Types of nosocomial infections	Bloodstream infection	26 (38.2%)	1.22 (0.63, 2.34)	
	Surgical site infection	31 (33.7%)	1.00	

Prognosis of the infections	Good	15 (16.3%)	1.00	1.00
	Poor	42 (61.8%)	8.23 (3.96, 17.35)	10.97 (4.57, 26.36)^*∗*^

Good prognosis indicates recovery without complications, poor prognosis indicates recovery with complications or death, COR indicates the crude odds ratio, and AOR indicates the adjusted odds ratio. ^*∗*^*P* value <0.05 was considered statistically significant.

## Data Availability

The datasets used and/or analyzed during this study are available from the corresponding author and provided upon reasonable request.

## Abbreviations

AOR: Adjusted odds ratio
CDC: Centers for Disease Control and Prevention
CI: Confidence interval
CLSI: Clinical Laboratory Standards Institute
COR: Crude odds ratio
ICU: Intensive care unit
MRSA: Methicillin-resistant *Staphylococcus aureus*
PCR: Polymerase chain reaction
SOP: Standard operating procedures
SPSS: Statistical Package for Social Sciences.
